# Supplementary material for: Bioaccessibility, Intestinal Absorption and Anti-Inflammatory Activity of Curcuminoids Incorporated in Avocado, Sunflower, and Linseed Beeswax Oleogels
Source: Foods. 2024 Jan 24;13(3):373. doi: 10.3390/foods13030373 (PMC10855298; doi:10.3390/foods13030373)

Table S1. Fatty acid composition of linseed, sunflower and avocado oils, expressed as percentage of methyl ester.

Fatty acid	Linseed oil (%)	Sunflower oil (%)	Avocado oil (%)
**C16:0**	5.23 ± 0.10	5.67 ± 0.03	13.40 ± 0.05
**C18:0**	3.61 ± 0.02	3.92 ± 0.10	0.56 ± 0.01
**C16:1**	-	-	4.42 ± 0.01
**C18:1n-9**	19.64 ± 0.12	41.98 ± 0.10	67.86 ± 0.17
**C18:2n-6**	14.51 ± 0.11	48.50 ± 0.10	12.55 ± 0.03
**C18:3n-3**	57.02 ± 0.25	0.17 ± 0.02	1.20 ± 0.21
**∑SFA**	8.83 ± 0.02	9.59 ± 0.01	13.96 ± 0.05
**∑MUFA**	19.64 ± 0.12	41.79 ± 0.07	72.29 ± 0.18
**∑PUFA**	71.53 ± 0.14	48.46 ± 0.08	13.75 ± 0.23
**∑PUFA/∑SFA**	8.10 ± 0.03	5.07 ± 0.01	1.00 ± 0.01

SFA: saturated fatty acids; MUFA: monounsaturated fatty acids; PUFA: polyunsaturated fatty acids

Figure S1. Desirability function for OG formulation (a: OGL; b: OGS; c: OGA).

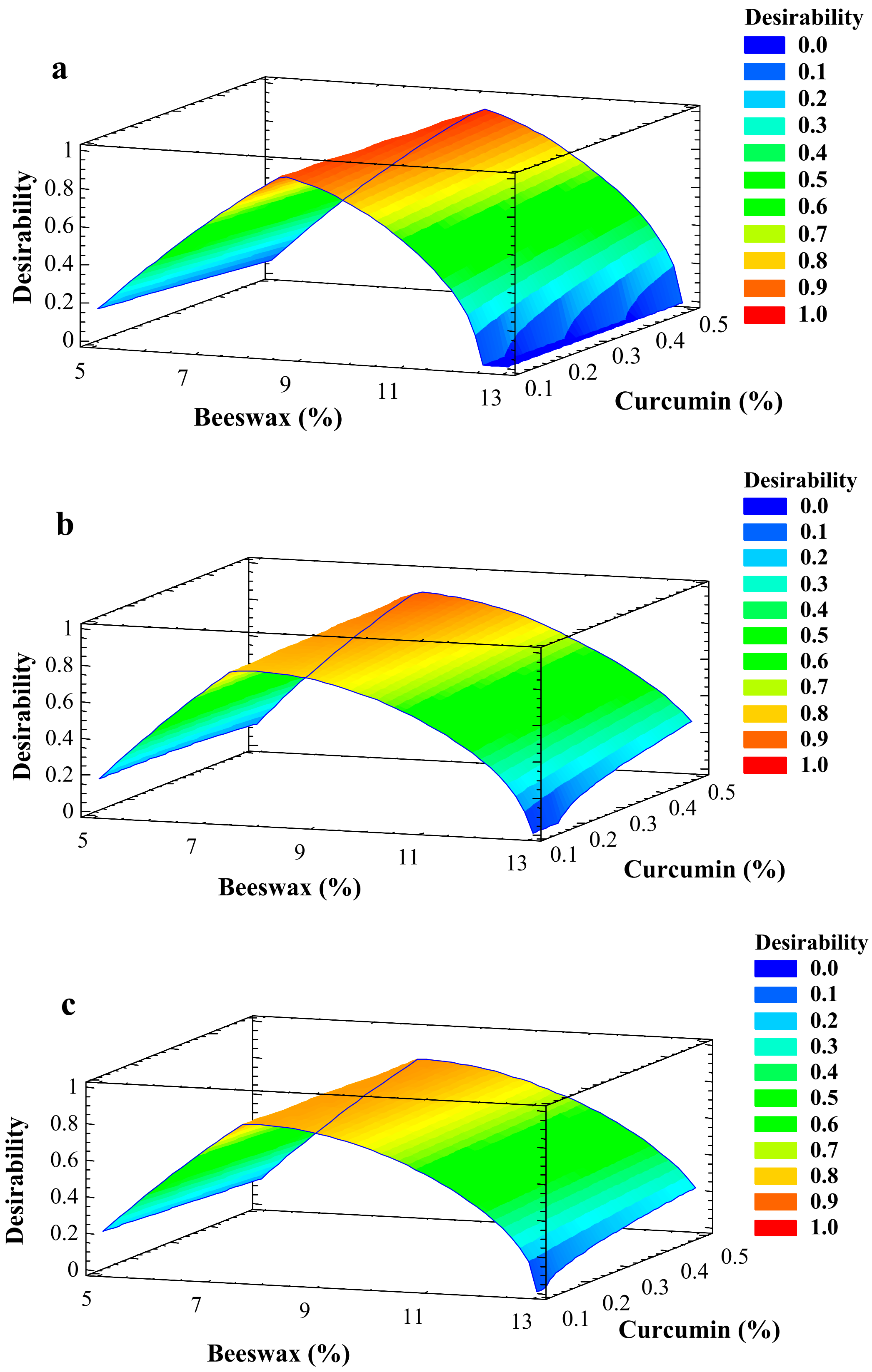

Figure S2. Typical HPLC chromatogram of curcuminoids. Component 1: curcumin, component 2: demethoxycurcumin, component 3: bisdemethoxycurcumin.

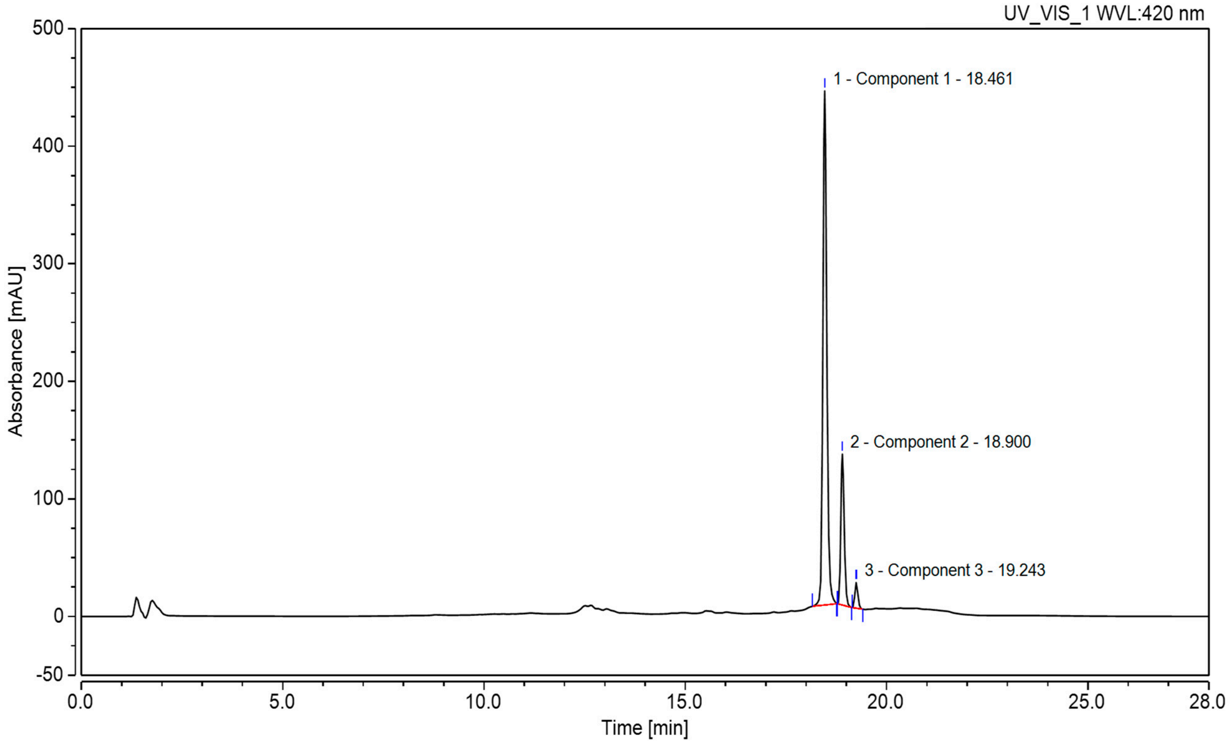

Figure S3. G' and G'' as a function of frequency for OG with curcumin (0.2% w/w; OGLCur, OGSCur and OGACur).

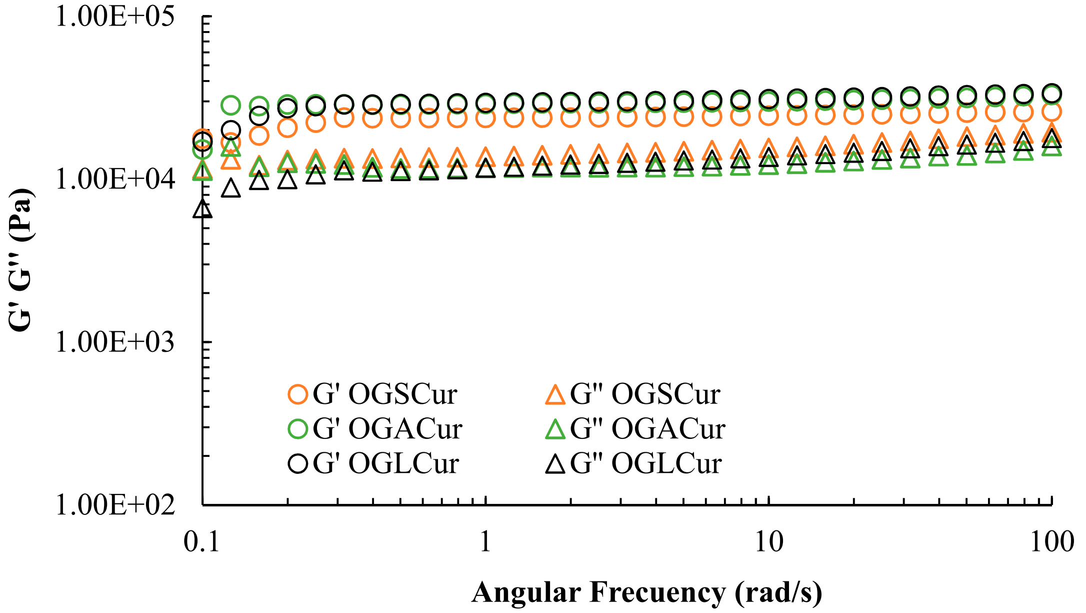

Figure S4. G’ and G’’ of OGLCur, OGSCur and OGACur as a function of temperature during heating from 10 °C to 70 °C.

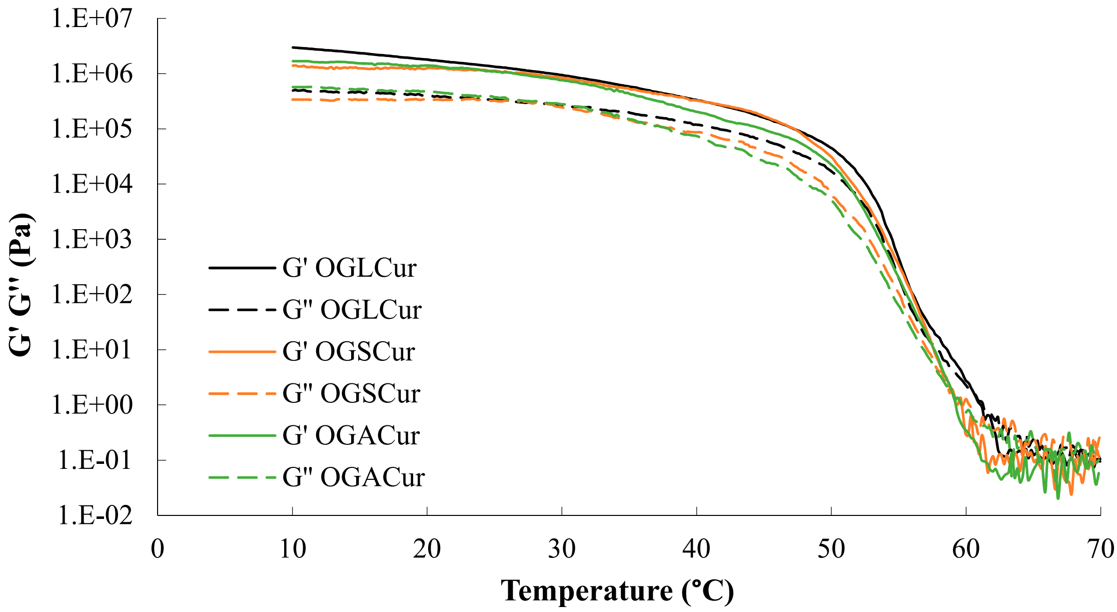

Figure S5. Cell viability of CaCo-2 (a) and ThP-1 (b) after 4 h and 24 h, respectively, of incubation with micellar phases from digested samples with curcumin (0.2% w/w).

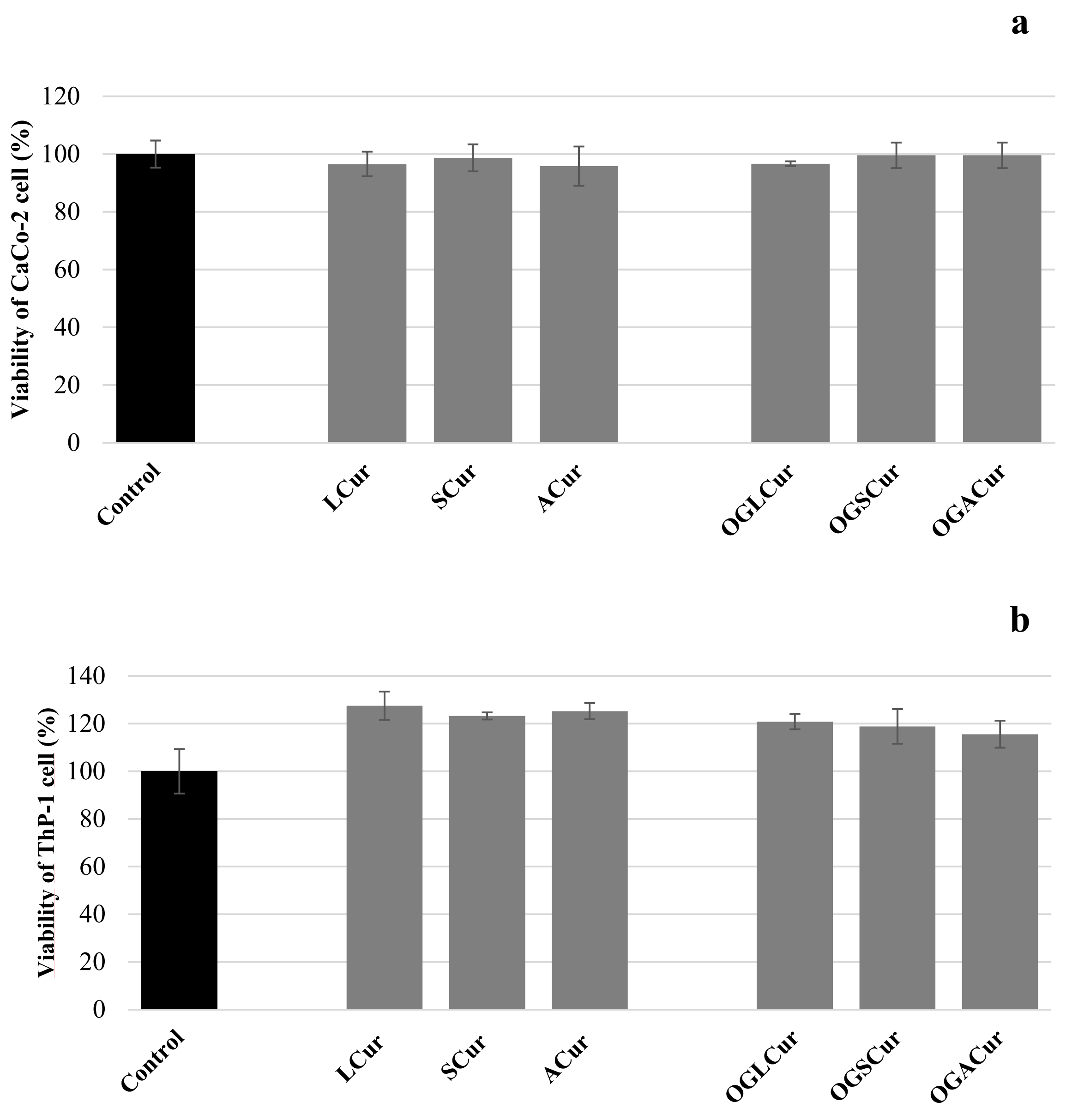

Supplement: Supplementary file 1 [file foods-13-00373-s001.zip › foods-2780742-supplementary.pdf]
